# Results of a hospitalization policy of asymptomatic and pre-symptomatic COVID-19-positive long-term care facility residents in the province of Salzburg—a report from the AGMT COVID-19 Registry

**DOI:** 10.1007/s11357-021-00352-y

**Published:** 2021-04-10

**Authors:** Florian Huemer, Gabriel Rinnerthaler, Benedikt Jörg, Patrick Morre, Birgit Stegbuchner, Elisabeth Proksch, Stefanie Fleimisch, Hannes Oberkofler, Iris Kremser, Richard Greil, Alexander Egle

**Affiliations:** 1 Department of Internal Medicine III with Haematology, Medical Oncology, Haemostaseology, Infectiology and Rheumatology, Oncologic Center, Salzburg Cancer Research Institute - Laboratory for Immunological and Molecular Cancer Research (SCRI-LIMCR), Paracelsus Medical University Salzburg, 5020 Salzburg, Austria; 2 Cancer Cluster Salzburg, 5020 Salzburg, Austria; 3 Department of Geriatric Medicine, Christian-Doppler-Klinik, Paracelsus Medical University Salzburg, 5020 Salzburg, Austria; 4 Department of Pneumology, Paracelsus Medical University Salzburg, 5020 Salzburg, Austria; 5 Department of Laboratory Medicine, Paracelsus Medical University Salzburg, 5020 Salzburg, Austria; 6 AGMT, 5020 Salzburg, Austria

**Keywords:** SARS-CoV-2, Pandemic, COVID-19, Long-term care facility, Nursing facility, Residents

## Abstract

COVID-19-associated case fatality rates up to 48% were reported among nursing facility residents. During the first wave of the COVID-19 pandemic, routine SARS-CoV-2 testing in long-term care facilities in the Province of Salzburg and centralized hospitalization in the COVID-19 unit of the Paracelsus Medical University Salzburg (Austria) irrespective of symptoms was implemented. Baseline characteristics and the course of COVID-19 disease were assessed among hospitalized long-term care facility residents within the COVID-19 Registry of the Austrian Group Medical Tumor Therapy (AGMT; NCT04351529). Between the 24^th^ of March and the 20^th^ of April 2020, 50 COVID-19-positive residents were hospitalized. The median age was 84.5 years (range: 79–88) and the median number of comorbidities and baseline medication classes was 6 (IQR: 4–7) and 5 (IQR: 3–6), respectively. At admission, 31 residents (62%) were symptomatic, nine residents (18%) pre-symptomatic whereas ten residents (20%) remained asymptomatic. The 30-day mortality rate from hospitalization was 32% and significantly higher in symptomatic residents at admission when compared to asymptomatic residents including pre-symptomatic residents (48% [95% CI: 27–63%] versus 5% [95% CI: 0–15%], *p=*0.006). The Early Warning Score (EWS) at admission was associated with 30-day mortality: high risk: 100%, intermediate risk: 50% (95% CI: 0–78%), and low risk: 21% (95% CI: 7-32%) (*p*<0.001). In light of comparably low mortality rates between asymptomatic and pre-symptomatic hospitalized COVID-19-positive residents, we suggest the supply of comparable intensity and quality of monitoring and care in long-term care facilities as an alternative to immediate hospitalization upon a positive COVID-19 test in asymptomatic residents.

**Supplementary Information:**

The online version contains supplementary material available at 10.1007/s11357-021-00352-y.

## Introduction

Coronavirus disease 2019 (COVID-19) is an infectious disease caused by severe acute respiratory syndrome coronavirus 2 (SARS-CoV-2). The disease was first identified at the end of 2019 in Wuhan, the capital of Hubei province in central China, and has since spread globally [1]. COVID-19 leads to high morbidity and mortality, mainly in elderly and comorbid populations [2], but late-sequelae may additionally burden younger patients, including previously healthy people. COVID-19 outbreaks in residential homes as well as in short- and long-term care facilities represent a considerable threat with hospitalization rates ranging from 4% in the UK [3] to 19% in the USA [4]. With case fatality rates up to 48% [3–6], reported COVID-19-associated deaths in nursing facilities have been unacceptably high. It is noteworthy that in some countries, COVID-19-associated deaths were not counted if death occurred outside the hospital (including long-term care facilities) [7]; therefore, death rates in long-term-care facilities might be underrecognized and underestimated. The Center for Disease Control and Prevention (CDC) updates its recommendations for COVID-19 infection prevention and control in nursing facilities [8] on a regular basis aiming at preventing the spread and protecting residents as well as health care workers from severe infection, hospitalization, and death. Similar to many other regions, long-term care facilities were ill-prepared for such a pandemic crisis. They were short of place for single person isolation, suffered training for use of personal protective equipment (PPE), which they were dramatically short of, and did not have granted regular, continuous, and acute care by practitioners trained for this situation. As a result, our policy was to hospitalize symptomatic as well as asymptomatic COVID-19 positive long-term care facility residents in the COVID-19 unit of the Paracelsus Medical University Salzburg (Austria) in order to allow infection control in sometimes very large residencies as well as to take over treatment of many very difficult to care for patients with substantial morbidities including neuropsychiatric symptoms or behavioral alterations. Despite the rollout of various COVID-19 vaccines [9–11], the implementation of vaccination strategies and prioritization of high-risk groups and health care personnel, factors such as e.g. shortness of supply and the occurrence of SARS-CoV-2 variants [12–14], could pose a considerable challenge to pandemic control in long-term care facilities. Also, very elderly and comorbid populations similar to long-term care residents were severely underrepresented in vaccine trials. Efficacy data may be very different in such a population and it is likely that it may be significantly lower.

In this single-center study, we aimed at studying the course of COVID-19 and clinical outcome in symptomatic and asymptomatic hospitalized long-term care facility residents in the province of Salzburg (Austria) included in the COVID-19 Registry of the Austrian Group Medical Tumor Therapy (AGMT, NCT04351529).

## Patients and methods

### Patients

In this observational analysis, we included unselected consecutively hospitalized residents of long-term care facilities (including nursing homes and facilities for mentally and/or physically handicapped people) that had been tested COVID-19 positive by RT-PCR from a nasopharyngeal swab. All included patients alive at the date of analysis gave their informed consent to participate in the AGMT COVID-19 Registry (NCT04351529), which has been documented in the respective medical chart. Due to the non-interventional nature of the AGMT COVID-19 Registry, only routine data, which have already been recorded in the patients’ medical charts, were analyzed. Symptoms during the preceding 14 days were assessed on an interview and review of medical records basis. Treatment indication, the decision to offer treatment, treatment choice, dose, schedule, and dose reductions/escalations were exclusively based on the risk/benefit estimation of the treating physician.

### RT-PCR

In the COVID-19 unit, RT-PCR tests (Altona Diagnostics, Germany) for SARS-CoV-2 were routinely performed from nasopharyngeal swabs.

### Discharge policy

Relief of COVID-19 associated symptoms for at least 48 h and two serial negative RT-PCR tests with a minimum interval of 24 h was a prerequisite for discharge from the COVID-19 unit. When the Austrian health ministry (paralleling the German Robert-Koch Institute [15]) issued new guidance allowing to use a quantitative marker (Ct above 30) to exclude infectious risk, the latter discharge policy was adopted.

### Early Warning Score

The Early Warning Score (EWS) incorporating respiratory rate, oxygen saturation, demand for oxygen supply, body temperature, systolic blood pressure, heart rate, and level of consciousness was applied for standard assessment of acute illness severity. A higher score reflects a more severe acute illness with EWS 0-4, EWS 5-6, and EWS ≥7 classified as low, intermediate, and high score, respectively (Supplementary Table 1) [16]. The frequency of assessment as well as the respective measures was based on the discretion of the treating physician.

### Patient categorization

Residents were classified as symptomatic if they had typical symptoms of a respiratory infection (with or without fever) such as cough, sore throat, shortness of breath, or atypical symptoms such as diarrhea, headache, fatigue, chills, myalgia, painful joints, or deterioration of confusion at the time point of admission to the COVID-19 unit. Residents without symptoms at admission to the COVID-19 unit, who developed typical or atypical symptoms during the course of disease, were classified as pre-symptomatic. Asymptomatic patients did never experience typical or atypical symptoms during medical care in the COVID-19 unit.

### Monitoring and treatment

Fluid balance charts, laboratory tests, blood gas analyses, and imaging studies were ordered on an individual basis. Medical supportive treatment was initiated at the discretion of the treating physician and mainly consisted of oxygen supply, administration of antibiotics, intravenous (iv) hydration, enteral and/or parenteral nutrition support, and physical and respiratory therapy. Low-molecular-weight heparin-based prophylactic anticoagulation therapy was temporarily established in each resident without pre-existing anticoagulation therapy. Access to treatment with the anti-interleukin-6 (IL-6) receptor blocking monoclonal antibody tocilizumab (400 mg iv up to two applications in total) was made available by Roche® explicitly for off-label use. The decision to apply tocilizumab was based on the oxygen demand and inflammation parameters on an individual basis. Remdesivir, dexamethasone, and convalescent plasma transfusions were not regularly applied during this study period.

### Statistical analyses

Differences in patient baseline characteristics between two groups (symptomatic versus asymptomatic; survivors versus non-survivors) were tested by Pearson’s *χ*^*2*^-test. For continuous data, the difference between the two groups was calculated with two-sided Wilcoxon rank-sum test. In an exploratory analysis, we used the Kaplan–Meier method for survival curves and to evaluate OS differences according to baseline characteristics. Log-rank test was used to compare survival distributions between two patient groups. A Fine–Gray regression model was used for competing risk analysis (death versus discharge). All analyses were performed using the statistical software environment R (version 3.5.1) including package “survival.”

## Results

During the first wave between March 2020 and April 2020, 50 residents from twelve long-term care facilities were tested COVID-19 positive by RT-PCR from nasopharyngeal swabs and hospitalized in the COVID-19 unit of the Paracelsus Medical University Salzburg (Austria) irrespective of symptoms. The median age of the entire cohort was 84.5 years and with a female (68%) to male (32%) preponderance (Table 1).

**Table 1:** Baseline characteristics, clinical symptoms, and comorbidities among 50 long-term care facility residents admitted to the COVID-19 unit who were hospitalized due to symptomatic or asymptomatic COVID-19 infection

	Total (*n*=50)	Symptomatic (*n*=31, 62%)	Asymptomatic (*n*=19, 38%)	*p* value
Age (median, IQR)	84.5 (79–88)	85 (79–87)	84 (76.5–89.5)	0.834*
Sex				0.500
Female	34 (68)	20 (65)	14 (74)	
Male	16 (32)	11 (35)	5 (26)	
EWS at admission (median, IQR)	3 (1–4)	3 (3–5)	1 (0–2)	<0.001*
New or increased oxygen demand at admission				0.006
No	30 (60)	14 (45)	16 (84)	
Yes	20 (40)	17 (55)	3 (16)	
New or increased oxygen demand at COVID-19 unit				
No	18 (37)	7 (23)	11 (61)	0.012
Yes	31 (63)	24 (77)	7 (39)	
Unknown	1	0	1	
ICU transfer				0.721
No	48 (96)	30 (97)	18 (95)	
Yes	2 (4)	1 (3)	1 (5)	
Clinical symptoms at admission				
Fever (> 37·5°C)				
No		11 (35)		
Yes		20 (65)		
Dyspnea				
No		15 (48)		
Yes		16 (52)		
Cough				
No		15 (48)		
Yes		16 (52)		
Headache				
No		31 (100)		
Yes		0 (0)		
Chills				
No		30 (97)		
Yes		1 (3)		
Sore throat				
No		30 (100)		
Yes		0 (0)		
Missing		1		
Rhinorrhea				
No		31 (100)		
Yes		0 (0)		
Diarrhea				
No		30 (97)		
Yes		1 (3)		
Fatigue				
No		18 (58)		
Yes		13 (42)		
Myalgia/painful joints				
No		30 (97)		
Yes		1 (3)		
Deteriorated confusion				
No		24 (77)		
Yes		7 (23)		
Comorbidities				
Number of comorbidities	6 (4–7)	5 (4–7)	6 (5–7)	0.179
Chronic lung disease				0.802
No	44 (88)	27 (87)	17 (90)	
Yes	6 (12)	4 (13)	2 (10)	
Cardiac disease^#^				0.425
No	22 (44)	15 (48)	7 (37)	
Yes	28 (56)	16 (52)	12 (63)	
Hypertension				0.163
No	7 (14)	6 (19)	1 (5)	
Yes	43 (86)	25 (81)	18 (95)	
Diabetes mellitus type 2				0.566
No	34 (68)	22 (71)	12 (63)	
Yes	16 (32)	9 (29)	7 (37)	
Cerebrovascular disease				0.392
No	36 (72)	21 (68)	15 (79)	
Yes	14 (28)	10 (32)	4 (21)	
Vascular disease				
No	45 (90)	29 (94)	16 (84)	0.285
Yes	5 (10)	2 (6)	3 (16)	
Chronic kidney disease				0.608
No	26 (52)	17 (55)	9 (47)	
Yes	24 (48)	14 (45)	10 (53)	
Neurodegenerative disease				0.491
No	38 (78)	22 (73)	16 (84)	
Yes	11 (22)	8 (27)	3 (16)	
Unknown	1	1	0	
Cognitive impairment				0.182
No	6 (12)	2 (7)	4 (21)	
Yes	42 (88)	27 (93)	15 (79)	
Unknown	2	2	0	
Active hematologic disease				
No	49 (98)	30 (97)	19 (100)	0.429
Yes	1 (2)	1 (3)	0 (0)	
Active oncologic disease				0.606
No	46 (92)	29 (94)	17 (90)	
Yes	4 (8)	2 (6)	2 (10)	
Thyroid disorder				0.968
No	37 (74)	23 (74)	14 (74)	
Yes	13 (26)	8 (26)	5 (26)	
History of thromboembolic events				0.660
No	41 (82)	26 (84)	15 (79)	
Yes	9 (18)	5 (16)	4 (21)	
Autoimmune disease				NA
No	50 (100)	31 (100)	19 (100)	
Yes	0 (0)	0 (0)	0 (0)	

*Wilcoxon rank-sum test

^#^Coronary heart disease, chronic heart failure, arrhythmia, and/or heart valve disease

*ICU*, intensive care unit; *NA*, not available; *EWS*, Early Warning Score

### Clinical symptoms

While COVID-19 testing was initially performed due to symptoms in 28 residents (56%), 22 residents (44%) were asymptomatic during SARS-CoV-2 screening tests in the long-term care facilities. At admission to the COVID-19 unit, 31 residents (62%) presented with typical or atypical symptoms and were classified “symptomatic.” Ten residents (20%) remained asymptomatic during the entire hospital stay whereas nine residents (18%) developed symptoms during the course of disease, classified as “pre-symptomatic.” Among symptomatic residents, 29 (94%) displayed typical COVID-19 symptoms whereas only two patients (6%) presented with atypical symptoms at admission.

### Comorbidities

Pre-existing comorbidities were present in the majority of residents with cognitive impairment (88%), hypertension (86%), cardiac disease including coronary heart disease, chronic heart failure, arrhythmia and/or heart valve disease (56%), chronic kidney disease (48%), and diabetes mellitus type II (32%) ranking among the leading comorbidities. The distribution of comorbidities did not statistically significantly differ between symptomatic and asymptomatic residents at admission. The median number of pre-existing comorbidities was 6 (IQR: 4–7) (Table 1).

### Co-medication

Psychopharmacologic drugs (68%), ACE inhibitors (30%), angiotensin receptor blockers (ARBs, 20%), and other antihypertensive agents (66%), proton pump inhibitors (52%), therapeutic anticoagulation therapy (34%), opiates (32%), and platelet aggregation inhibitors (26%) were the most frequently reported co-medication classes. Statistically significant differences of pre-existing co-medication were found between symptomatic and asymptomatic residents at admission to the COVID-19 unit: ACE inhibitors (19% versus 47%, *p*=0.036), ARBs (29% versus 5%, *p*=0.041), antibiotics (19% versus 0%, *p*=0.041), and opiates (19% versus 53%, *p*=0.014). The median number of baseline medication classes was 5 (IQR: 3–6) (Table 2).

**Table 2:** Co-medication and laboratory values of 50 long-term care facility residents hospitalized due to symptomatic or asymptomatic COVID-19 infection

	Total (*n*=50)	Symptomatic (*n*=31, 62%)	Asymptomatic (*n*=19, 38%)	*p* value
Co-medication at admission				
Number of medication classes	5	4	5	0.255*
	(3–6)	(3–6)	(4–6)	
ACE inhibitor				0.036
No	35 (70)	25 (81)	10 (53)	
Yes	15 (30)	6 (19)	9 (47)	
ARBs				0.041
No	40 (80)	22 (71)	18 (95)	
Yes	10 (20)	9 (29)	1 (5)	
Other antihypertensive therapy				0.130
No	17 (34)	13 (42)	4 (21)	
Yes	33 (66)	18 (58)	15 (79)	
Antibiotics				0.041
No	44 (88)	25 (81)	19 (100)	
Yes	6 (12)	6 (19)	0 (0)	
Proton pump inhibitors				0.944
No	24 (48)	15 (48)	9 (47)	
Yes	26 (52)	16 (52)	10 (53)	
Statins				0.287
No	38 (76)	22 (71)	16 (84)	
Yes	12 (24)	9 (29)	3 (16)	
NSAIDs				0.123
No	44 (88)	29 (94)	15 (79)	
Yes	6 (12)	2 (6)	4 (21)	
Opiates				0.014
No	34 (68)	25 (81)	9 (47)	
Yes	16 (32)	6 (19)	10 (53)	
Long-term systemic steroid therapy (≥14 days)				0.197
No	49 (98)	31 (100)	18 (95)	
Yes	1 (2)	0 (0)	1 (5)	
Short-term systemic steroid therapy (<14 days)				0.429
No	49 (98)	30 (97)	19 (100)	
Yes	1 (2)	1 (3)	0 (0)	
Inhaled steroid therapy				0.162
No	47 (94)	28 (90)	19 (100)	
Yes	3 (6)	3 (10)	0 (0)	
Antidiabetic therapy (excluding insulin)				0.579
No	43 (86)	26 (84)	17 (90)	
Yes	7 (14)	5 (16)	2 (10)	
Insulin therapy				0.519
No	44 (88)	28 (90)	16 (84)	
Yes	6 (12)	3 (10)	3 (16)	
Psychopharmacologic therapy				0.500
No	16 (32)	11 (35)	5 (26)	
Yes	34 (68)	20 (65)	14 (74)	
Anticoagulation therapy (therapeutic dose)^§^				0.344
No	33 (66)	22 (71)	11 (58)	
Yes	17 (34)	9 (29)	8 (42)	
Antiplatelet therapy				0.481
No	37 (74)	24 (77)	13 (68)	
Yes	13 (26)	7 (23)	6 (32)	
Laboratory values (IQR)				
CRP (mg/dl)	4.8 (1.5–12.0)	4.6 (1.9–10.3)	5.2 (1.35–13.7)	0.764*
Peak CRP (mg/dl)	10.1 (4.4–16.3)	9.8 (4.5–15.6)	12.2 (4.4–17.2)	0.849*
IL-6 (pg/ml)	36.9 (20.8–102)	37.5 (20.9–100.3)	36.9 (17–96.4)	0.774*
Peak IL-6 (pg/ml)	72.9 (33.1–231)	81.7 (35–206.8)	72.9 (34.3–258)	0.975*
Ferritin (mcg/l)	426.5 (196–910)	537 (324.5–1150)	226 (149.5–453.5)	0.019*
Peak ferritin (mcg/l)	540.5 (300.5–1323)	890 (430.5–1610)	398 (236–672.5)	0.009*
Procalcitonin (mcg/l)	0.1 (0.1–0.2)	0.1 (0.1–0.3)	0.1 (0.1–0.2)	0.262*
Peak procalcitonin (mcg/l)	0.2 (0.1–0.5)	0.3 (0.1–0.5)	0.2 (0.1–0.5)	0.578*
ATIII (%)	87 (77–100)	90 (79–101)	83 (75.5–96.5)	0.448*
D-dimer (mg/l)	1.02 (0.65–2.80)	1.79 (0.82–3.65)	0.73 (0.57–1.16)	0.015*
Fibrinogen (mg/dl)	443 (346–483)	443 (375.5–479.5)	431 (338–476)	0.644*
Prothrombin time (%)	75 (68–88)	76.5 (66.5–89)	75 (68.5–82.5)	0.805*
PTT (s)	35 (31–38)	35 (31–39.5)	34 (31.5–36)	0.673*
LDH (U/l)	279 (209.5–312)	281 (218–329)	267 (209.5–302)	0.413*
Hs troponin T (ng/l)	36 (24–49)	40.5 (34–65)	28 (22–44.5)	0.149*
Creatine kinase (U/l)	70 (37–154)	105 (61.5–157)	51 (35–137)	0.186*
GOT (U/l)	31 (25–48)	34.5 (27–55)	30 (22–36.5)	0.107*
GPT (U/l)	23 (15–33)	25.5 (17–35.5)	22 (12.5–27)	0.134*
Creatinin (mg/dl)	1.13 (0.82–1.59)	1.17 (0.83–1.88)	1 (0.83–1.13)	0.156*
eGFR (ml/min/BSA)	49.5 (32–67)	46 (27–67)	58 (41–66.5)	0.213*
Blood urea nitrogen (mg/dl)	46.5 (35–75.5)	52 (37.5–97.5)	41 (32.5–49.5)	0.032*
Na^+^ (mmol/l)	139 (136–144)	140 (136–145)	138 (136–141)	0.363*
Platelets (G/l)	187 (150–251)	181 (151–238)	195 (157–260)	0.912*
Hemoglobin (g/dl)	12.2 (11–13.6)	12.7 (11.6–13.8)	11.6 (10.6–12.9)	0.039*
WBC (G/l)	5.54 (4.32–7.88)	5.81 (4.12–7.41)	5.23 (4.54–8.69)	0.639*
ANC (G/l)	3.67 (2.81–6.02)	3.87 (2.66–5.82)	3.55 (3.13–6.97)	0.378*
ALC (G/l)	0.85 (0.62–1.15)	0.87 (0.62–1.17)	0.79 (0.64–1.04)	0.750*

*Wilcoxon rank-sum test

^§^vitamin K antagonists, new oral anticoagulants or low-molecular-weight heparin

*ACE*, angiotensin-converting enzyme; *ALC*, absolute lymphocyte count; *ANC*, absolute neutrophil count; *ARB*, angiotensin receptor blocker; *BSA*, body surface area; *CRP*, C-reactive protein; *eGFR*, estimated glomerular filtration rate; *GOT*, glutamate-oxaloacetate transaminase; *GPT*, glutamine phenylpyruvate transaminase; *IL-6*, interleukin 6; *LDH*, lactate dehydrogenase; *NSAID*, non-steroidal anti-inflammatory drug; *PTT*, partial thromboplastin time; *WBC*, white blood cell count

### Baseline laboratory values

Symptomatic residents at admission showed significantly higher baseline levels of ferritin (537 versus 226 mcg/l, *p*=0.019), D-dimer (1.79 versus 0.73 mg/l, *p*=0.015), blood urea nitrogen (BUN, 52 versus 41 mg/dl, *p*=0.032), and hemoglobin (12.7 versus 11.6 g/dl, *p*=0.039) when compared to asymptomatic residents (Table 2).

### COVID-19-directed treatment

Tocilizumab was applied in six patients due to COVID-19 pneumonia with respiratory deterioration and laboratory signs of hyperinflammation. Dexamethasone was initiated due to COVID-19 pneumonia in one patient. None of the residents received remdesivir, hydroxychloroquine, or convalescent plasma.

### Length of hospital stay

The median length of hospital stay was 21 days (IQR: 7–29) in the overall population and 27 days (IQR: 21–34) in patients without in-hospital death. Asymptomatic residents and symptomatic residents at admission could be discharged after a median of 27 days from the COVID-19 unit (*p*=0.66).

### Discharge probability and 30-day mortality rate

**Fig. 1:**
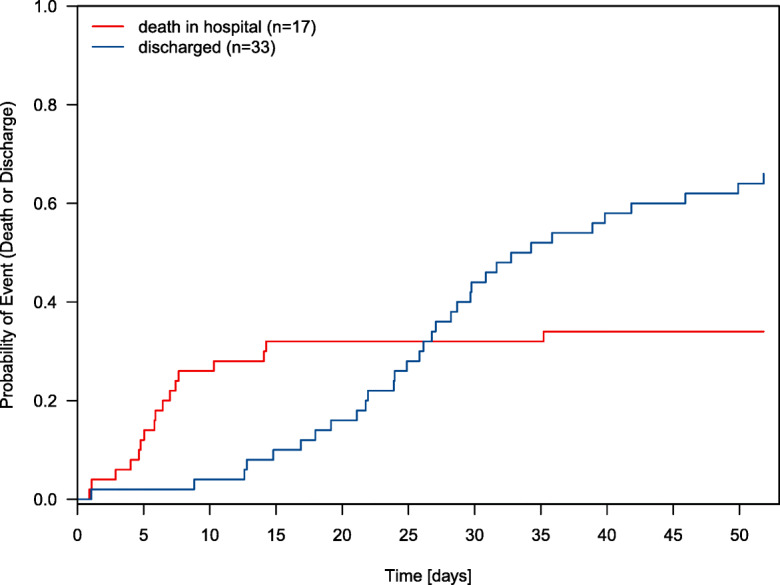
Cumulative incidence of discharge or death among 50 long-term care facility residents. *y*-axis: probability of discharge or death, *x*-axis: time in days from admission

**Fig. 2:**
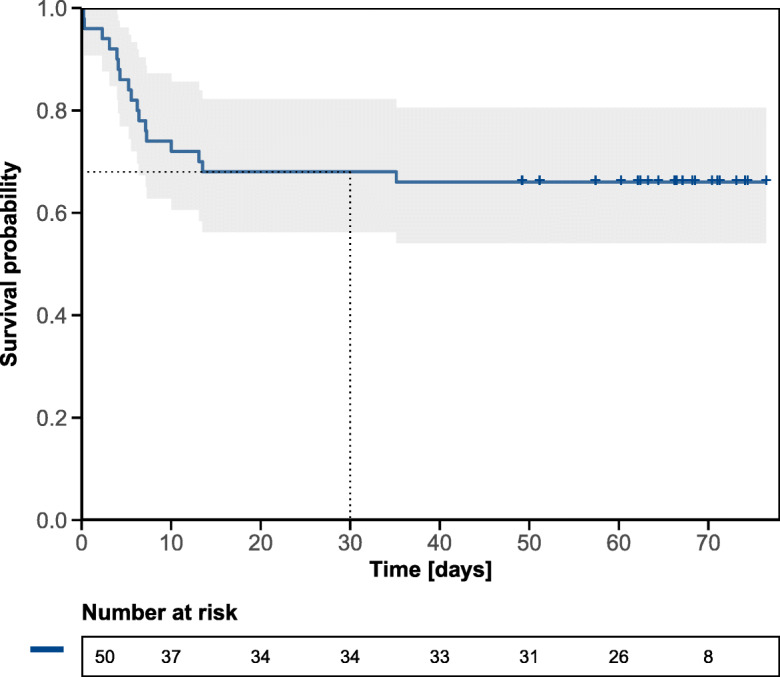
Overall survival from admission to the COVID-19 unit among 50 long-term care facility residents. *y*-axis: survival probability, *x*-axis: time in days from admission. Tick marks on the curve represent censored patients

**Fig. 3:**
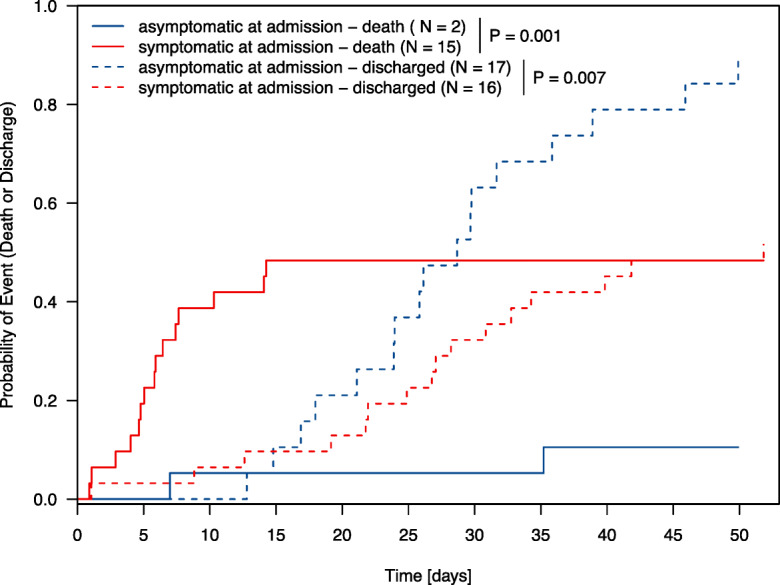
Cumulative incidence of discharge or death according to COVID-19 symptoms at hospital admission. *y*-axis: cumulative death or discharge probability, *x*-axis: time in days from admission

**Fig. 4:**
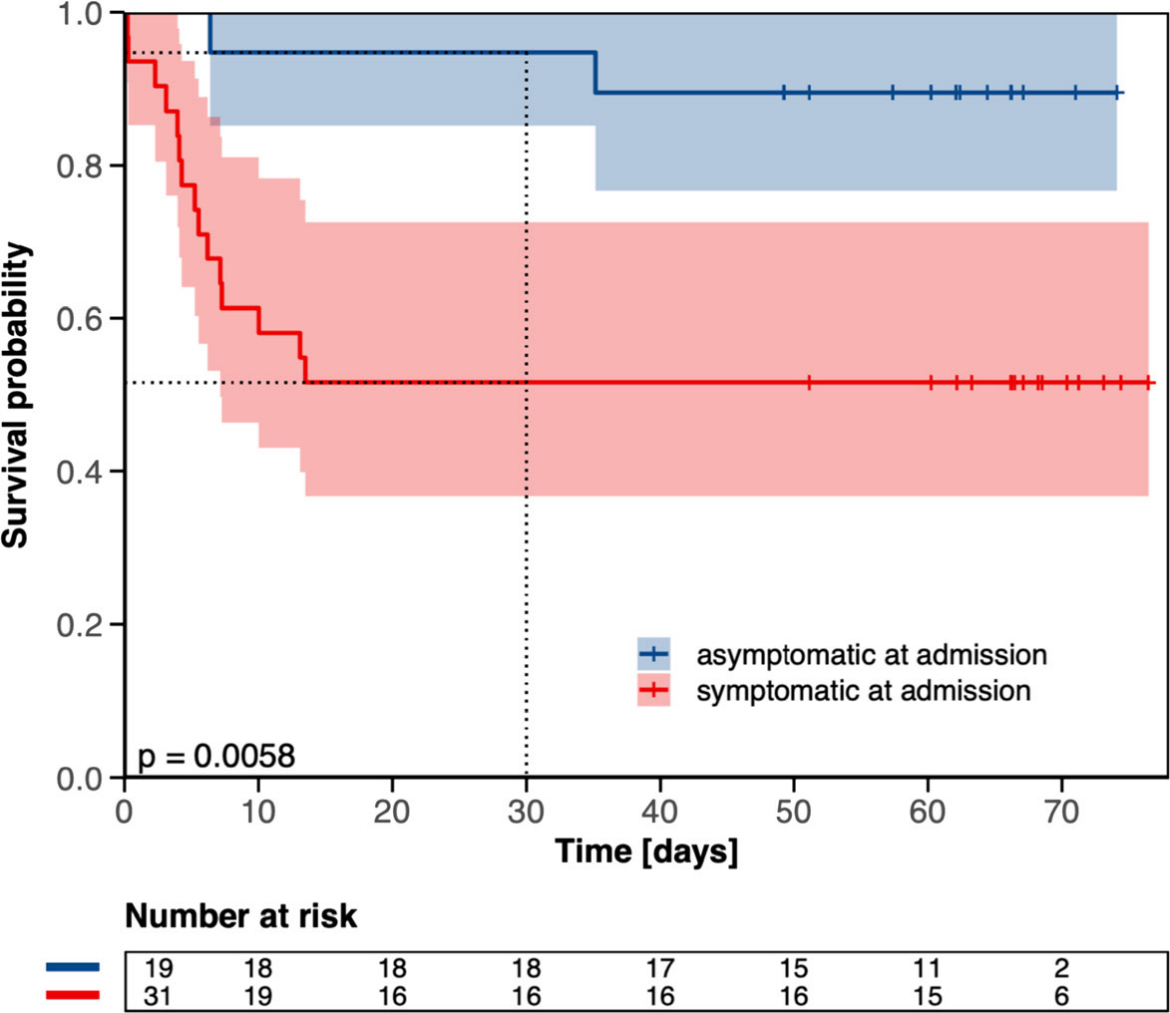
Overall survival according to COVID-19 symptoms at hospital admission among 50 long-term care facility residents. *y*-axis: survival probability, *x*-axis: time in days from admission. Tick marks on the curves represent censored patients; dashed vertical line depicts 30-day cut-off

**Fig. 5:**
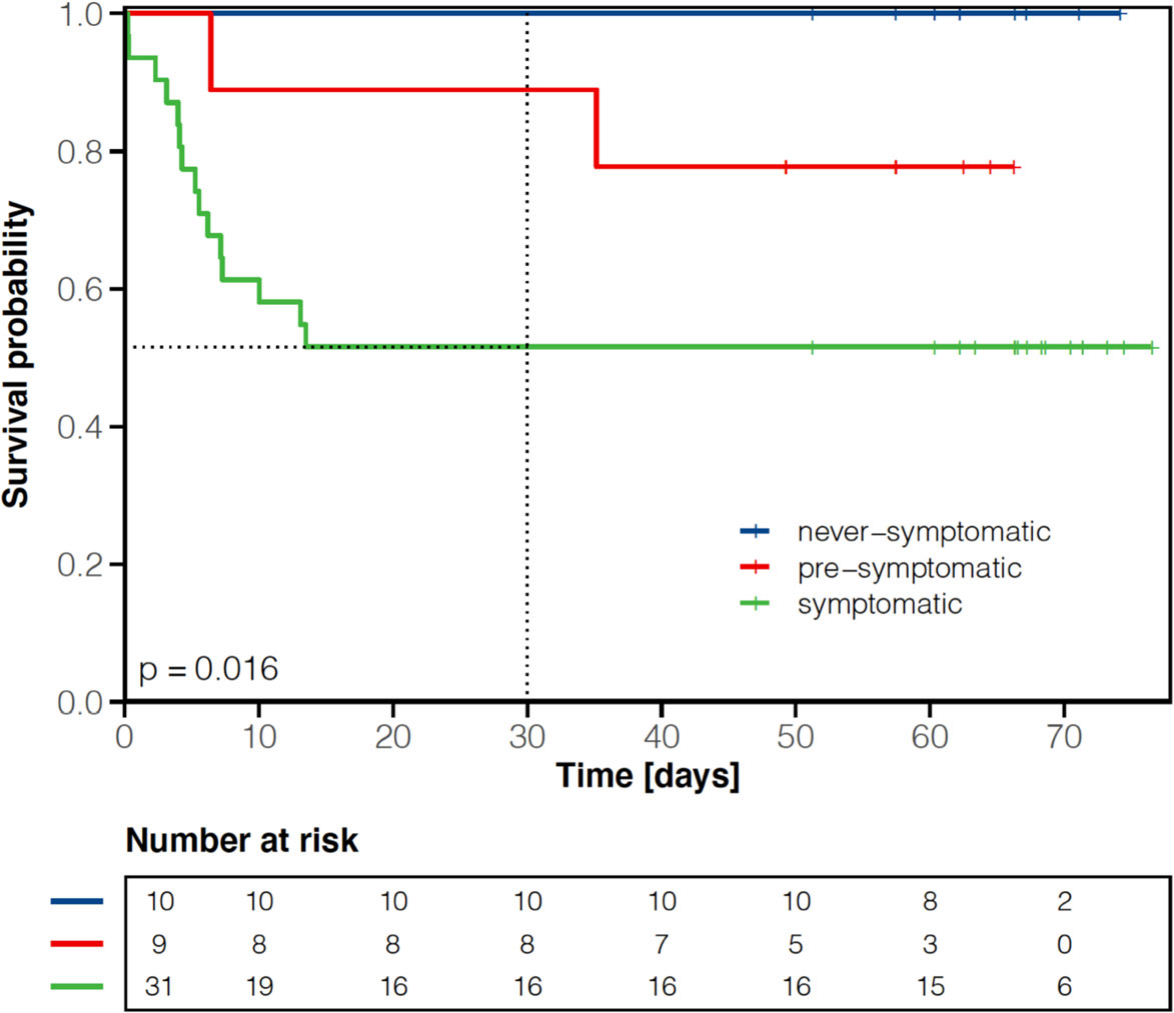
Overall survival according to COVID-19 symptoms during the course of disease among 50 long-term care facility residents. *y*-axis: survival probability, *x*-axis: time in days from admission. Tick marks on the curves represent censored patients; dashed vertical line depicts 30-day cut-off

Although the median time to the first negative SARS-CoV-2 RT-PCR from nasopharyngeal swabs was 11 days, it took a median of 17 days until documentation of two serial negative nasopharyngeal swabs. In total, two patients were transferred to the intensive care unit (ICU) due to respiratory deterioration. While 33 residents (66%) could be discharged from the COVID-19 unit to the long-term care facilities or to another non-infectious ward, 17 patients (34%) succumbed to the COVID-19 infection (Fig. 1). The 30-day mortality rate from hospitalization was 32% (Fig. 2). One patient died from COVID-19-associated cardiovascular complications on another medical ward after release from quarantine. The cumulative discharge and death probability according to the presence or absence of clinical symptoms at admission are depicted in Fig. 3. Overall survival from admission to the COVID-19 unit in symptomatic residents was statistically significantly worse compared to asymptomatic residents including pre-symptomatic residents (median not reached in both groups, HR 6.18 [95% CI: 1.41–27.07], *p*=0.02 Cox proportional hazard model, Fig. 4). The presence/absence of COVID-19-associated symptoms at admission as well as the development of symptoms during the hospital stay had a statistically significant impact on survival (*p*=0.016 log-rank, Fig. 5).

### Comparison of baseline characteristics, comorbidities, co-medication, and laboratory values between survivors and non-survivors

The time interval between symptom onset and RT-PCR testing was statistically significantly shorter among survivors compared to non-survivors (median: 0 days versus 1.5 days, *p*=0.029). While 94% of non-survivors developed a new or increased oxygen demand during the course of disease, this was only the case in 48% of survivors (*p*=0.006). Fever (69% versus 38%, *p*=0.044), fatigue (63% versus 18%, *p*=0.002), and new onset or deterioration of confusion (37% versus 12%, *p*=0.034) at admission to the COVID-19 unit were more frequently documented in non-survivors in comparison to survivors. The frequency of cognitive impairment was higher among residents that succumbed to COVID-19 (100% versus 82%, *p*=0.029). Higher baseline levels of ferritin (800.5 versus 337.5 mcg/l, *p*=0.013), procalcitonin (0.2 versus 0.1, *p*=0.018), D-dimer (2.8 versus 0.87, *p=*0.007), LDH (303 versus 243 U/l, *p*=0.041), GOT (51 versus 30 U/l, *p*=0.003), plasma sodium (142.5 versus 138 mmol/l, *p*=0.014), and blood urea nitrogen (76 versus 39.5 mg/dl, *p*<0.001) were measured in non-survivors compared to survivors whereas baseline estimated glomerular filtration rate (eGFR) was statistically significantly higher in survivors (55 versus 33 ml/min/BSA, *p*=0.019) (Table 3).

**Table 3:** Baseline characteristics according to 30-day mortality of 50 long-term care facility residents admitted to the COVID-19 unit

	Total (*n*=50)	Dead (*n*=16, 32%)	Alive (*n*=34, 68%)	*p* value
Age (median, IQR)	84.5 (79–88)	85.5 (77–87)	83.5 (79–89)	0.819*
Sex				0.567
Female	34 (68)	10 (63)	24 (71)	
Male	16 (32)	6 (37)	10 (29)	
EWS at admission (median, IQR)	3 (1–4)	4 (3–6)	2 (0–3)	<0.001*
New or increased oxygen demand at admission				0.108
No	30 (60)	7 (44)	23 (68)	
Yes	20 (40)	9 (56)	11 (32)	
New or increased oxygen demand at COVID-19 unit				0.006
No	18 (37)	1 (6)	17 (52)	
Yes	31 (63)	15 (94)	16 (48)	
Unknown	1	0	1	
Time from symptom onset to SARS-CoV-2 test (days)	0 (–1–4)	1.5 (0–4)	0 (–3–2)	0.029*
ICU transfer				0.578
No	48 (96)	15 (94)	33 (97%)	
Yes	2 (4)	1 (6)	1 (3)	
Clinical symptoms at admission				
Fever (> 37.5°C)				0.044
No	26 (52)	5 (31)	21 (62)	
Yes	24 (48)	11 (69)	13 (38)	
Dyspnea				0.827
No	27 (54)	9 (56)	18 (53)	
Yes	23 (46)	7 (44)	16 (47)	
Cough				0.960
No	31 (62)	10 (63)	21 (62)	
Yes	19 (38)	6 (37)	13 (38)	
Headache				NA
No	50 (100)	16 (100)	34 (100)	
Yes	0 (0)	0 (0)	0 (0)	
Chills				0.141
No	49 (98)	15 (94)	34 (100)	
Yes	1 (2)	1 (6)	0 (0)	
Sore throat				NA
No	49 (100)	15 (100)	34 (100)	
Yes	0 (0)	0 (0)	0 (0)	
Unknown	1	1	0	
Rhinorrhea				NA
No	50 (100)	16 (100)	34 (100)	
Yes	0 (0)	0 (0)	0 (0)	
Diarrhea				0.959
No	47 (94)	15 (94)	32 (94)	
Yes	3 (6)	1 (6)	2 (6)	
Fatigue				0.002
No	40 (80)	6 (37)	28 (82)	
Yes	10 (20)	10 (63)	6 (18)	
Myalgia/painful joints				0.141
No	49 (98)	15 (94)	34 (100)	
Yes	1 (2)	1 (6)	0 (0)	
Deteriorated confusion				0.034
No	40 (80)	10 (63)	30 (88)	
Yes	10 (20)	6 (37)	4 (12)	
Comorbidities				
Number of comorbidities (median, IQR)	6 (4–7)	5 (4–6)	6 (4–7)	0.462
Chronic lung disease				0.391
No	44 (88)	15 (94)	29 (85)	
Yes	6 (12)	1 (6)	5 (15)	
Cardiac disease^#^				0.981
No	22 (44)	7 (44)	15 (44)	
Yes	28 (56)	9 (56)	19 (56)	
Hypertension				0.834
No	7 (14)	2 (12)	5 (15)	
Yes	43 (86)	14 (88)	29 (85)	
Diabetes mellitus type 2				0.467
No	34 (68)	12 (75)	22 (65)	
Yes	16 (32)	4 (25)	12 (35)	
Cerebrovascular disease				0.726
No	36 (72)	11 (69)	25 (74)	
Yes	14 (28)	5 (31)	9 (26)	
Vascular disease				0.106
No	45 (90)	16 (100)	29 (85)	
Yes	5 (10)	0 (0)	5 (15)	
Chronic kidney disease				0.680
No	26 (52)	9 (56)	17 (50)	
Yes	24 (48)	7 (44)	17 (50)	
Neurodegenerative disease				0.716
No	38 (78)	13 (81)	25 (76)	
Yes	11 (22)	3 (19)	8 (24)	
Unknown	1	0	1	
Cognitive impairment				0.029
No	6 (12)	0 (0)	6 (18)	
Yes	42 (88)	14 (100)	28 (82)	
Unknown	2	2	0	
Active hematologic disease				0.141
No	49 (98)	15 (94)	34 (100)	
Yes	1 (2)	1 (6)	0 (0)	
Active oncologic disease				0.421
No	46 (92)	14 (88)	32 (94)	
Yes	4 (8)	2 (12)	2 (6)	
Thyroid disorder				0.912
No	37 (74)	12 (75)	25 (74)	
Yes	13 (26)	4 (25)	9 (26)	
History of thromboembolic events				0.925
No	41 (82)	13 (81)	28 (82)	
Yes	9 (18)	3 (19)	6 (18)	
Autoimmune disease				NA
No	50 (100)	16 (100)	34 (100)	
Yes	0 (0)	0 (0)	0 (0)	
Co-medication at admission				
Number of medication classes	5	4	5	0.291*
(median, IQR)	(3–6)	(3–5)	(4–6)	
ACE inhibitor				0.597
No	35 (70)	12 (75)	23 (68)	
Yes	15 (30)	4 (25)	11 (32)	
ARBs				0.544
No	40 (80)	12 (75)	28 (82)	
Yes	10 (20)	4 (25)	6 (18)	
Other antihypertensive therapy				0.720
No	17 (34)	6 (37)	11 (32)	
Yes	33 (66)	10 (63)	23 (68)	
Antibiotics				0.314
No	44 (88)	13 (81)	31 (91)	
Yes	6 (12)	3 (19)	3 (9)	
Proton pump inhibitors				0.846
No	24 (48)	8 (50)	16 (47)	
Yes	26 (52)	8 (50)	18 (53)	
Statins				0.910
No	38 (76)	12 (75)	26 (77)	
Yes	12 (24)	4 (25)	8 (23)	
NSAIDs				0.391
No	44 (88)	15 (94)	29 (85)	
Yes	6 (12)	1 (6)	5 (15)	
Opiates				0.168
No	34 (68)	13 (81)	21 (62)	
Yes	16 (32)	3 (19)	13 (38)	
Long-term systemic steroid therapy (≥14 days)				0.488
No	49 (98)	16 (100)	33 (97)	
Yes	1 (2)	0 (0)	1 (3)	
Short-term systemic steroid therapy (<14 days)				0.488
No	49 (98)	16 (100)	33 (97)	
Yes	1 (2)	0 (0)	1 (3)	
Inhaled steroid therapy				0.220
No	47 (94)	16 (100)	31 (91)	
Yes	3 (6)	0 (0)	3 (9)	
Antidiabetic therapy (excluding insulin)				0.507
No	43 (86)	13 (81)	30 (88)	
Yes	7 (14)	3 (19)	4 (12)	
Insulin therapy				0.391
No	44 (88)	15 (94)	29 (85)	
Yes	6 (12)	1 (6)	5 (15)	
Psychopharmacologic therapy				0.567
No	16 (32)	6 (37)	10 (29)	
Yes	34 (68)	10 (63)	24 (71)	
Anticoagulation therapy (therapeutic dose)^§^				0.357
No	33 (66)	12 (75)	21 (62)	
Yes	17 (34)	4 (25)	13 (38)	
Antiplatelet therapy				0.912
No	37 (74)	12 (75)	25 (74)	
Yes	13 (26)	4 (25)	9 (26)	
Laboratory values (IQR)				
CRP (mg/dl)	4.8 (1.5–12)	7.4 (3.6–13.2)	4.6 (1.4–10.9)	0.253*
Peak CRP (mg/dl)	10.1 (4.4–16.3)	11.6 (5.4–16.9)	9 (4.3–16)	0.355*
IL-6 (pg/ml)	36.9 (20.8–102)	63.3 (23.4–137.5)	30.4 (14.3–80.8)	0.067*
Peak IL-6 (pg/ml)	72.9 (33.1–231)	145 (59.1–279)	61.4 (29.2–182)	0.159*
Ferritin (mcg/l)	426.5 (196–910)	800.5 (436.5–1211.5)	337.5 (156–550)	0.013*
Peak ferritin (mcg/l)	540.5 (300.5–1323)	1255 (514.5–2079)	445.5 (274–852.5)	0.006*
Procalcitonin (mcg/l)	0.1 (0.1–0.2)	0.2 (0.1–0.6)	0.1 (0.1–0.1)	0.018*
Peak procalcitonin (mcg/l)	0.2 (0.1–0.5)	0.4 (0.2–0.9)	0.1 (0.1–0.4)	0.025*
ATIII (%)	87 (77–99.5)	82 (76–98)	88 (79–103)	0.548*
D-dimer (mg/l)	1.02 (0.65–2.80)	2.8 (1.26–5.95)	0.87 (0.59–1.68)	0.007*
Fibrinogen (mg/dl)	443 (346–483)	449.5 (420.5–487)	409 (338–469)	0.364*
PTZ (%)	75 (68–88)	72.5 (63–84)	79 (69–88)	0.557*
PTT (s)	35 (31–38)	36.5 (32–41)	34 (31–36)	0.325*
LDH (U/l)	279 (209.5–312)	303 (281–372)	243 (191.5–309)	0.041*
Hs troponin T (ng/l)	36 (24–49)	46 (34.5–66.5)	36 (23–48)	0.187*
Creatine kinase (U/l)	70 (37–154)	98 (81.5–359.5)	61.5 (37–141.5)	0.088*
GOT (U/l)	31 (25–48)	51 (32–69)	30 (22–37)	0.003*
GPT (U/l)	23 (15–33)	29 (16–36)	22 (14–32)	0.182*
Creatinin (mg/dl)	1.13 (0.82–1.59)	1.55 (0.98–3.30)	1.03 (0.82–1.22)	0.052*
eGFR (ml/min/BSA)	49.5 (32–67)	33 (15.5–54.5)	55 (41–68)	0.019*
Blood urea nitrogen (mg/dl)	46.5 (35–75.5)	76 (51–116)	39.5 (29.5–51.5)	<0.001*
Na^+^ (mmol/l)	139 (136–144)	142.5 (139–150)	138 (135–142)	0.014*
Platelets (G/l)	187 (150–251)	150 (142–216)	196 (170–270)	0.060*
Hemoglobin (mg/dl)	12.2 (11–13.4)	12.6 (11.1–13.6)	12.2 (11–13.4)	0.662*
WBC (G/l)	5.54 (4.32–7.88)	6.73 (4.68–8.21)	5.15 (4.16–7.76)	0.303*
ANC (G/l)	3.67 (2.81–6.02)	4.99 (3.40–6.59)	3.38 (2.74–5.59)	0.168*
ALC (G/l)	0.85 (0.62–1.15)	0.8 (0.58–1.14)	0.87 (0.65–1.14)	0.441*

*Wilcoxon rank-sum test

^#^Coronary heart disease, chronic heart failure, arrhythmia, and/or heart valve disease

^§^Vitamin K antagonists, new oral anticoagulants, or low-molecular-weight heparin

*ACE*, angiotensin-converting enzyme; *ALC*, absolute lymphocyte count; *ANC*, absolute neutrophil count; *ARB*, angiotensin receptor blocker; *BSA*, body surface area; *CRP*, C-reactive protein; *eGFR*, estimated glomerular filtration rate; *GOT*, glutamate-oxaloacetate transaminase; *GPT*, glutamine phenylpyruvate transaminase; *IL-6*, interleukin 6; *LDH*, lactate dehydrogenase; *NSAID*, non-steroidal anti-inflammatory drug; *PTT*, partial thromboplastin time; *WBC*, white blood cell count

### Early Warning Score and association with clinical outcome

**Fig. 6:**
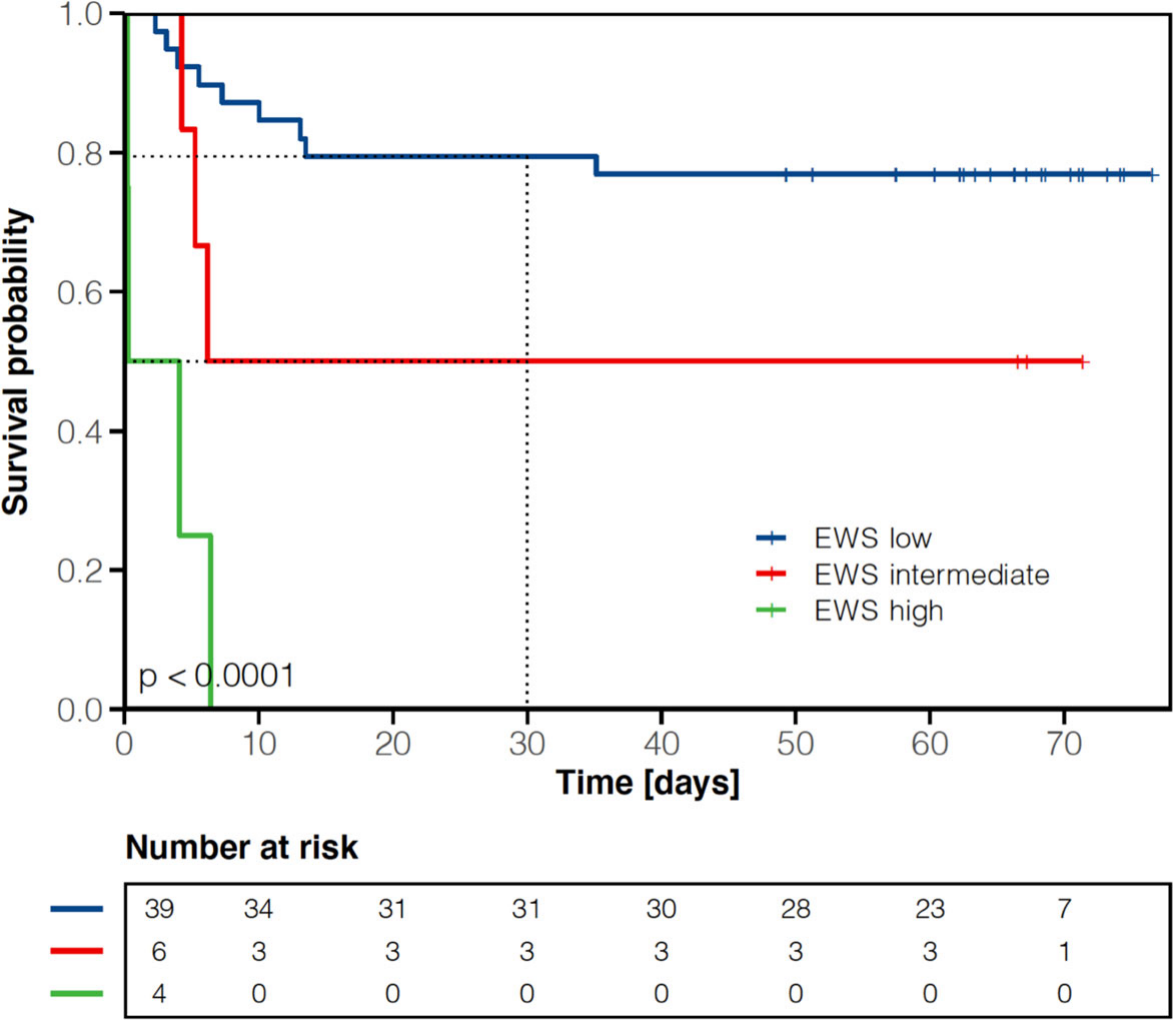
Overall survival according to baseline EWS among 49 long-term care facility residents with available EWS. *y*-axis: survival probability, *x*-axis: time in days from admission. Tick marks on the curves represent censored patients; dashed vertical line depicts 30-day cut-off

The EWS at admission to the COVID-19 unit was available in 49 residents. The baseline EWS turned out to be prognostic for the 30-day mortality rate among COVID-19-positive residents: EWS high risk: 100%, EWS intermediate risk: 50% (95% CI: 0–78%), and EWS low risk: 21% (95% CI: 7–32%), *p*<0.001 (Fig. 6). Compared to a low-risk EWS (0–4 points), a high-risk EWS (≥7 points) was associated with a worse overall survival (HR 17.92 [95% CI 4.84–66.37], *p*<0.001). No statistically significant difference was observed between the intermediate (5–6 points) and low-risk group (HR 2.83 [95% CI 0.76–10.53], *p*=0.12).

## Discussion

People of advanced age and particular residents of long-term care facilities face a high death toll and excess mortality from COVID-19 in most countries in the world [17]. Several strategies have been proposed to selectively protect this extremely vulnerable population from infection, severe morbidity, and death. Unfortunately, however, such approaches were either not implemented or not successful during the first wave and apparently not even during the second wave of the pandemic 8 to 9 months later, despite much better availability of PPE and testing capacity, some effective medication, and even better medical experience with the disease. The approval status of COVID-19 vaccines for elderly people as well as the progress of COVID-19 vaccination programs for high-risk groups such as long-term care facility residents is highly variable between various countries and parts of the world. These issues raise the question how to better deal with these vulnerable cohorts.

To the best of our knowledge, this is the first investigation of a hospitalization policy of COVID-19-positive long-term care facility residents irrespective of symptoms. The approach to hospitalize symptomatic and asymptomatic COVID-19-positive residents in the Province of Salzburg enabled us to closely monitor the course of disease in this geriatric and comorbid cohort in a well-equipped setting. In our cohort, the median length of hospital stay was 21 days (IQR: 7–29) in the overall population and 27 days (IQR: 21–34) in patients without in-hospital death. One out of three hospitalized residents succumbed to the COVID-19 infection, corroborating the high mortality rate in this vulnerable population [3–6]. Notably, the majority of deaths were derived from symptomatic residents at admission (Figs. 3, 4, and 5). In our cohort, roughly every other asymptomatic resident at admission developed COVID-19-associated symptoms during the course of the hospital stay. We thus confirm that asymptomatic SARS-CoV-2 infection is seen in a relevant percentage even in elderly and comorbid residents of long-term care facilities [18]. However, due to diagnoses often made during screening efforts tracking local outbreaks, we observed a significant number of pre-symptomatic patients, being diagnosed very early in their course of disease and with a relatively low likelihood of a strong selection bias.

Although this has not been tested in a controlled, prospective manner, we observed a comparably encouraging clinical outcome in pre-symptomatic and asymptomatic residents by applying the following measures during the hospital stay.

Medical rounds were scheduled on a daily regular basis with the possibility of repeated visits according to the patients’ disease severity and demands. Due to the frequently observed at least temporary immobility and the procoagulant state associated with a COVID-19 infection [19, 20], all hospitalized residents received anticoagulation therapy at least in a prophylactic dose. Hyposmia or anosmia [21] as well as delirium [22] has been regularly reported among SARS-CoV-2-infected people and in turn may result in inadequate nutrient and fluid intake especially among elderly people. Metabolomics analyses demonstrated an altered amino acid and fatty acid metabolism in patients suffering from COVID-19 when compared to COVID-19-negative controls [23]. In this regard, the fluid balance was assessed on an individual basis and residents were provided with enteral and/or temporary parenteral nutrition support in case of reduced fluid or nutrient intake. Repeated laboratory testing, blood gas analyses, and imaging studies enabled us to rapidly detect imminent, potentially life-threatening complications such as kidney or respiratory failure and in turn to prompt timely counteractions such as e.g. intravenous fluid support, antibiotic therapy, or transfer to the ICU without delay. Physical therapy was offered to all residents; residents with pulmonary involvement were additionally supported by respiratory therapy. However, due to the limited number of pre-symptomatic residents (*n*=9), we cannot fully exclude a more indolent course of COVID-19 disease in the pre-symptomatic cohort when compared to symptomatic residents, irrespective of the abovementioned measures.

While 40% of residents presented with a new or increased oxygen demand at admission to the COVID-19 unit, the percentage rose to 60% during the hospital stay. However, owed to patients’ will, advance directives, and comorbidities, only two out of 50 residents were transferred to the ICU. In the meanwhile, study results of several COVID-19 directed therapies have been published. Remdesivir received approval by the Food and Drug Administration for COVID-19-positive hospitalized patients irrespective of disease severity [24, 25], but the large WHO Solidarity Trial could not prove an OS or disease-modifying effect [26]. In contrast, dexamethasone (in case of reduced oxygen saturation or demand for oxygen therapy) provided evidence for a substantial OS benefit in patients hospitalized for COVID-19 [27]. We emphasize that dexamethasone and remdesivir, for which clinical phase III trials have demonstrated an improved clinical outcome in the meanwhile, were not routinely or not applied at all in this cohort, respectively. Based on preliminary released efficacy data [28], tocilizumab was applied in six patients with evidence of respiratory failure and laboratory signs of hyperinflammation of which three residents succumbed to their COVID-19 pneumonia. Although the EMPACTA trial met its primary endpoint, demonstrating a reduced likelihood of needing mechanical ventilation in hospitalized patients with COVID-19 pneumonia [29], a plethora of clinical phase III trials did not show a survival benefit or other clear clinical benefits [30–32] of tocilizumab in COVID-19 disease. These findings together with Roche’s recent announcement of negative results from the phase III COVACTA trial [33] and a press release with positive results from the RECOVERY trial [34] shows that the question about the role of IL-6 receptor blockade in severe COVID-19-associated pneumonia is not yet resolved.

While previous reports described an association between pre-existing coronary artery disease [35, 36], congestive heart failure[35], arrhythmia [35], diabetes [36], and chronic obstructive lung disease [35, 36], respectively, with in-hospital death in hospitalized COVID-19-positive patients, COVID-19 associated mortality was not impacted by cardiovascular disease, diabetes mellitus, or chronic obstructive lung disease in our analysis. However, fatigue, pre-existing cognitive impairment, and deterioration or new onset of confusion were associated with an increased likelihood of in-hospital death in this cohort (Table 3), suggesting that the latter symptoms might represent clinically meaningful warning signs in elderly COVID-19-positive people. In this regard, it is interesting to note that infection with SARS-CoV-2 has recently been associated with onset of delirium even in afebrile patients and SARS-CoV-2-associated delirium turned out to be statistically significantly associated with the likelihood of admission to the ICU as well as with death [22]. Similar observations have been made among hospitalized influenza patients in whom dementia was an independent risk factor for mortality [37]. Early published retrospective data from China did not show an association between the presence of fatigue and mortality among COVID-19-positive patients [36]. However, a considerable difference in median age exists between the latter cohort (56 years) and our geriatric cohort (84.5 years) and fatigue has been shown to significantly impact mortality in older adults [38].

Within this geriatric population, 82% of residents presented with pre-existing hypertension treated with ACE inhibitors or ARBs in 30% and 20%, respectively (Table 2). SARS-CoV-2 uses the SARS-CoV-2 receptor ACE2 for entry into humans cells [39], and the expression of ACE2 might be increased by ACE inhibitors and ARBs according to animal studies [40]. ACE inhibitor and ARB use were not associated with worse survival in our cohort (Table 3), which is in line with retrospective analyses [41] as well as with the results from the randomized, controlled BRACE-CORONA trial [42]. In contrast to previous reports [43], proton pump inhibitor intake did not impact survival among the long-term care facility residents included in our analysis, although proton pump inhibitors were prescribed in more than half of all residents (Table 3). In line with the literature [44], pre-established therapeutic anticoagulation therapy (with either vitamin K antagonists, new oral anticoagulants, or low-molecular-weight heparin) was equally distributed between survivors and non-survivors (Table 3). In our cohort, each resident received at least anticoagulation therapy in a prophylactic dose; however, whether the latter measure had a beneficial impact on clinical outcome cannot be answered from our data. Pre-established opiate therapy was associated with a lower probability of COVID-19-associated symptoms at admission (Table 2); however, this did not translate into superior OS (Table 3). Literature covering the impact of opiates on symptoms and clinical outcome in COVID-19 disease is lacking. The majority of residents (68%) received psychopharmacologic medication, including antidepressants. In this regard, it is interesting that the selective serotonin reuptake inhibitor fluoxetine has been shown to inhibit SARS-CoV-2 cell entry in the cell culture model [45]. However, the application of psychopharmacologic drugs did not impact clinical symptoms or clinical outcomes in our cohort.

Non-survivors showed statistically significantly higher baseline levels of acute-phase reactants (ferritin, D-dimer) and elevated levels of procalcitonin, suggestive of bacterial superinfection (Table 3). The latter findings in our geriatric cohort are in line with reports from COVID-19-positive hospitalized patients or outpatients in China [2]. Furthermore, higher levels of sodium, blood urea nitrogen, and a worse eGFR, suggestive of reduced fluid intake and dehydration, were observed in non-survivors at baseline (Table 3), arguing for the individual establishment of our recommended measures (fluid balance charts, i.v. hydration, enteral and/or parenteral nutrition support) in asymptomatic as well as pre-symptomatic COVID-19-positive long-term care facility residents in-house.

A statistically significantly longer time interval between symptom onset and RT-PCR testing was found among residents succumbing to COVID-19 disease when compared to survivors (median: 1.5 days versus 0 days, *p*=0.029), suggesting rapid RT-PCR testing in case of a clinically suspected COVID-19 infection and subsequent immediate hospitalization in case of test positivity in symptomatic residents.

In clinical practice, it is of utmost importance to rapidly and repeatedly assess the clinical condition in hospitalized COVID-19 patients, in particular, in case of high bed occupancy rates and limited health personnel. We could demonstrate the feasibility to routinely apply the EWS in order to repeatedly assess the severity of COVID-19 disease. The EWS is easily calculated in clinical practice within a short period of time with non-invasive measures (Supplementary Table 1). Assessment of the EWS at hospital admission was prognostic for clinical outcome in this COVID-19-positive long-term care facility cohort (Fig. 6); therefore, we recommend applying the EWS in hospitalized COVID-19-positive patients as well as in long-term care facilities in order to objectify disease severity and to prompt countermeasures.

The main limitation of our analysis is the limited number of 50 long-term care facility residents; therefore, conclusions from our findings have to be drawn with caution. Our proposed monitoring and therapeutic measures were associated with a low mortality rate among pre-symptomatic residents; however, a definitive causal role of these measures cannot be derived from our data. Certainly, the rollout of the COVID-19 vaccination strategies might contribute to a significant change in the prognosis and management of this vulnerable population. However, the successful vaccination of a large proportion of people around the world may face significant hurdles and take substantial time periods for which the problem addressed will persist at least in part.

## Conclusions

Case fatality rates among hospitalized long-term care facility residents were mainly derived from symptomatic residents at hospital admission. Deterioration or new onset of fatigue, confusion or fever, and laboratory signs of hyperinflammation, dehydration, or even renal failure were associated with an increased likelihood of COVID-19-associated death and therefore should prompt immediate hospitalization in this vulnerable cohort. Pre-symptomatic residents who developed symptoms during the hospital stay showed a comparably good clinical outcome as residents who remained asymptomatic during the course of disease. Based on the latter findings, we suggest the supply of comparable intensity and quality of monitoring and care for asymptomatic and pre-symptomatic COVID-19-positive long-term care facility residents in-house aiming at saving hospital resources.

## Supplementary information

ESM 1 (DOCX 13 kb)

## Data Availability

Data are available from the corresponding author on reasonable request.
