# Supplementary material for: Results of a hospitalization policy of asymptomatic and pre-symptomatic COVID-19-positive long-term care facility residents in the province of Salzburg—a report from the AGMT COVID-19 Registry
Source: GeroScience. 2021 Apr 10;43(4):1877–97. doi: 10.1007/s11357-021-00352-y (PMC8035610; doi:10.1007/s11357-021-00352-y)
Supplement: Supplementary file 1 — (DOCX 13 kb) [file 11357_2021_352_MOESM1_ESM.docx]

**Supplementary Table 1 – Early Warning Score**

| **Respiratory rate (BPM)** | | | | **Oxygen saturation (%)** | | | **Suppl. oxygen** | **Temperature (°C)** | | | | | **Systolic blood pressure (mmHg)** | | | | | | | **Heart rate (BPM)** | | | | | | **Level of consciousness** | | | | **EWS Score** |
| --- | --- | --- | --- | --- | --- | --- | --- | --- | --- | --- | --- | --- | --- | --- | --- | --- | --- | --- | --- | --- | --- | --- | --- | --- | --- | --- | --- | --- | --- | --- |
| >25 | 21-24 | 9-20 | ≤8  8 | ≥94 | 91-93 | ≤90 | yes | >39 | >38 | 36-38 | <36 | <35 | >220 | >200 | >180 | 110-180 | <110 | <100 | <90 | >130 | >110 | >100 | 100-50 | <50 | <40 | awake | verbal | pain | unresponsive |  |
| 3 | 2 | 0 | 3 | 0 | 2 | 3 | 1 | 2 | 1 | 0 | 1 | 3 | 3 | 2 | 1 | 0 | 1 | 2 | 3 | 3 | 2 | 1 | 0 | 1 | 3 | 0 | 1 | 3 | |  |
| **Score** | | | | | **Risk group** | | | |  | | | | | | | | | | | | | | | | | | | | | |
| 0-4 | | | | | low | | | |  |  |  |  |  |  |  |  |  |  |  |  |  |  |  |  |  |  |  |  |  |  |
| 5-6 | | | | | intermediate | | | |  |  |  |  |  |  |  |  |  |  |  |  |  |  |  |  |  |  |  |  |  |  |
| ≥7 | | | | | high | | | |  |  |  |  |  |  |  |  |  |  |  |  |  |  |  |  |  |  |  |  |  |  |

BPM: breaths per minute or beats per minute
